# Tyrosine Is Associated with Insulin Resistance in Longitudinal Metabolomic Profiling of Obese Children

**DOI:** 10.1155/2016/2108909

**Published:** 2015-12-21

**Authors:** Christian Hellmuth, Franca Fabiana Kirchberg, Nina Lass, Ulrike Harder, Wolfgang Peissner, Berthold Koletzko, Thomas Reinehr

**Affiliations:** ^1^Division of Metabolic and Nutritional Medicine, Dr. von Hauner Children's Hospital, Ludwig-Maximilians-University of Munich, Lindwurmstraße 4, 80337 Munich, Germany; ^2^Department of Pediatric Endocrinology, Diabetes and Nutrition Medicine, Vestische Hospital for Children and Adolescents, University of Witten-Herdecke, Dr. Friedrich Steiner Strasse 5, 45711 Datteln, Germany

## Abstract

In obese children, hyperinsulinaemia induces adverse metabolic consequences related to the risk of cardiovascular and other disorders. Branched-chain amino acids (BCAA) and acylcarnitines (Carn), involved in amino acid (AA) degradation, were linked to obesity-associated insulin resistance, but these associations yet have not been studied longitudinally in obese children. We studied 80 obese children before and after a one-year lifestyle intervention programme inducing substantial weight loss >0.5 BMI standard deviation scores in 40 children and no weight loss in another 40 children. At baseline and after the 1-year intervention, we assessed insulin resistance (HOMA index), fasting glucose, HbA1c, 2 h glucose in an oral glucose tolerance test, AA, and Carn. BMI adjusted metabolite levels were associated with clinical markers at baseline and after intervention, and changes with the intervention period were evaluated. Only tyrosine was significantly associated with HOMA (*p* < 0.05) at baseline and end and with change during the intervention (*p* < 0.05). In contrast, ratios depicting BCAA metabolism were negatively associated with HOMA at baseline (*p* < 0.05), but not in the longitudinal profiling. Stratified analysis revealed that the children with substantial weight loss drove this association. We conclude that tyrosine alterations in association with insulin resistance precede alteration in BCAA metabolism. This trial is registered with ClinicalTrials.gov Identifier NCT00435734.

## 1. Introduction

Obesity in childhood is strongly associated with cardiovascular risk factors (CRFs) including dyslipidemia, hyperglycaemia, and hypertension [1]. In obese children hyperinsulinaemia and other CRFs are far more commonly found than in normal weight children and adolescents [2–4]. Most metabolic consequences appear to be mediated through insulin resistance (IR) [5]; therefore improving insulin sensitivity seems even more important than weight loss [6]. “Omics” platforms, such as proteomics, transcriptomics, epigenomics, and metabolomics, provide insights into molecular changes and allow assessing biochemical alterations in the development of obesity and IR [7, 8]. While new targets or potential biomarkers are identified in humans with these approaches [9, 10], the role of known metabolites still needs to be evaluated. Particularly, the influence of amino acid (AA) metabolism on the onset of IR still needs clarification. Two recent studies have reported on the untargeted metabolomic approach to study the relation of metabolites to IR in older adults [11] and children [12]. Untargeted metabolomics involves an unbiased screening of all metabolites present in a specimen regardless of chemical class. Targeted metabolomic techniques facilitate the profiling of specific metabolites of interest in a given population, to aid in-depth analysis of metabolic processes in the context of preformed findings. Thus, clinical targeted metabolomics platforms are suitable tools to reveal associations between AA and IR. Different studies depicted associations between IR or type 2 diabetes mellitus (T2DM) and branched-chain amino acids (BCAA), aromatic amino acids (AAA), sulphur containing AA, and other AAs as well as short-chain acylcarnitines (Carn) involved in AA metabolism in adults [13–23]. BCAA were found to be positively associated with homeostasis model assessment (HOMA), an IR index, in nonobese Chinese men [15] and young Finn adults [16]. Mohorko et al. recently reported elevated serum levels of cysteine (Cys) and tyrosine (Tyr) as early biomarkers for metabolic syndrome in young adults [14]. Newgard et al. showed that BCAA and short-chain Carn derived from BCAA contribute to the development of obesity-associated IR [13]. However, the majority of these studies describe relations of clinical markers to metabolites in cross-sectional settings. Furthermore, such associations are susceptible to confounders, like dietary protein intake that was shown to be higher in obese subjects than in normal weight subjects [15]. A few studies describe the prediction potential of BCAA and AAA for the onset of IR [16, 18, 24]. Although metabolomic analyses in children yield the potential to investigate the early onset of metabolic disease, studies on obese children are lacking. Recently, Newbern et al. reported an association of HOMA with a metabolic signature containing BCAA, uric acid, and long-chain Carn in adolescent boys in a cross-sectional study [25]. A combination of BCAA and AAA was associated with HOMA in obese Hispanic children [26], but only BCAA in Korean children [27]. BCAA pattern and androgen hormone pattern were associated with childhood adiposity and cardiometabolic risk, like HOMA, in another recently published cross-sectional study [12]. Longitudinal studies are necessary to explore stronger association between IR and metabolic alterations. To our knowledge, two longitudinal studies in children have been published so far, showing an association of baseline BCAA with HOMA in healthy American children [28] and in Korean children [27].

We embarked on a longitudinal study on obese children participating in a lifestyle intervention for inducing weight loss to explore the relationship between changes in AA metabolism and IR in the fasting state and after an oral glucose tolerance test (oGTT) in obese European children. Additionally, we analysed the obesity-independent associations of changes during the intervention period in makers of IR, hemoglobin A1c (HbA1c), 2 h glucose in oGTT, and changes of AA and Carn.

## 2. Methods

### 2.1. Study

Written informed consent was obtained from all parents of the participants prior to inclusion in the study. The study has been performed according to the Declaration of Helsinki. The local ethics committee of the University of Witten/Herdecke in Germany approved the study (ClinicalTrials.gov Identifier NCT00435734). We studied 80 obese Caucasian children participating in the one-year lifestyle intervention “Obeldicks,” which has been described in detail elsewhere [29]. Briefly, this outpatient intervention program is based on promoting regular physical activity, nutrition education, and behavior therapy including individual psychological care of the children and their families. The one-year training program was divided into three phases. In the first one, intensive phase (3 months), the children took part in the nutritional course and in the eating-behavior course in six group-sessions, each lasting for 1.5 hours. Parents were invited to attend six evening classes. In the establishing phase (6 months), individual psychological family therapy was provided (30 minutes/month). In the last phase of the program (accompanying the families back to their everyday lives) (3 months), further individual care was possible, if and when necessary. None of the children in the current study were smokers, took any drugs, or suffered from endocrine disorders or syndromal obesity such as Prader Willi syndrome [30]. Also MC4 receptor mutation was excluded. The children studied were selected at random from the Obeldicks cohort reported previously [30] choosing 40 obese children with substantial weight loss and 40 obese children without weight loss of similar age, gender, pubertal stage, and degree of overweight. We included only children who participated in oGTT both at baseline and after one year. Substantial reduction of overweight was defined by a decrease in standard deviation score of body mass index (BMI-SDS) ≥ 0.5 based on previous studies [31], whereas no reduction of overweight was defined by a decrease in BMI-SDS < 0.15. The metabolomic profile of these children in respect to obesity status and weight loss was previously reported [32].

### 2.2. Measurements and Sampling

Height was measured to the nearest millimeter using a rigid stadiometer. Weight was measured unclothed to the nearest 0.1 kg using a calibrated balance scale. BMI was calculated as weight in kilograms (kg) divided by the square of height in meters (m^2^). The degree of overweight was quantified using Cole's LMS method, which normalized the BMI skewed distribution and expressed BMI as a standard deviation score (BMI-SDS) [33]. Reference data for German children were used [34]. Waist circumference was measured halfway between lower rib and iliac crest.

For longitudinal analysis, blood samples were collected in the fasting state before the intervention and after 1 year. Furthermore, oGTT were performed according to current guidelines [35]. The glucose load was 1.75 g/kg with a maximum of 75 g. Blood samples were taken at 8 a.m. after overnight fasting for at least 10 hours. Following coagulation at room temperature, blood samples were centrifuged for 10 min at 8000 rpm at room temperature and aliquoted. Glucose (Boehringer, Mannheim, Germany), HbA1c (Germany Tina-quant Hemoglobin A1c Gen), and insulin (Abbott, Wiesbaden, Germany) were measured in serum by using commercially available test kits directly. Intra-assay and interassay CVs of glucose, HbA1c, and insulin were less than 5%. HOMA was used to detect the degree of IR [36]. Furthermore, serum samples were stored at –81°C and thawed at room temperature for the metabolomics assay only once.

### 2.3. Biochemical Measures

Metabolites were qualified and quantified with the Absolute IDQ p 150 kit (Biocrates Life Sciences AG, Innsbruck, Austria) as described previously [32]. Briefly, 10 *μ*L of blood serum was analysed with a flow injection tandem mass spectrometer (FIA-MS/MS). An Agilent 1200 SL series high-performance liquid chromatography system (Agilent, Waldbronn, Germany) was coupled to a hybrid quadrupole mass spectrometer (QTRAP 4000, AB Sciex, Darmstadt, Germany). MS/MS analysis was run in Multiple Reaction Monitoring mode with electrospray ionization used in both positive and negative modes. Data acquisition on the mass spectrometer was controlled by Analyst 1.5 software (AB Sciex, Darmstadt, Germany). For raw data processing, peak integration, isotope correction, calibration, and quality control, the Met IQ software package (Biocrates Life Sciences AG, Innsbruck, Austria) was used, which is an integral part of the Absolute* IDQ* kit quantifying a total of 163 metabolites. Middle- and long-chain Carn, sphingomyelins (SM), acyl-linked phosphatidylcholines, ether-linked phosphatidylcholines, and lysophosphatidylcholines were not used for the data analysis of this work, since the presented study focused on alterations in AA metabolism with respect to IR. For the presented work, we analyzed 14 short-chain Carn (C*x*:*y*, hydroxyl acylcarnitines C*x*:*y*-OH, oxoacylcarnitines C*x*:*y*-oxo, and dicarboxylacylcarnitines C*x*:*y*-DC), free carnitine (Carn C0), and 14 AA. C*x*:*y* abbreviates the lipid side chain composition, *x* and *y* denoting the number of carbons and double bonds, respectively. The sum of leucine (Leu) and isoleucine (Ile) is expressed as xLeu. Samples were integrated with the Met IQ software by automated calculation of metabolite concentrations. For the data analysis performed here, only short-chain Carn, Carn C0, and AA are used. The sum of xLeu and valine (Val) is expressed as BCAA sum. The sum of phenylalanine (Phe), tryptophan (Trp), and Tyr is expressed as AAA sum. We report all metabolite concentrations in *μ*mol/L. In addition to the 29 metabolite concentrations and two sum parameters, eleven metabolite ratios were calculated resulting in a total of 42 metabolites and metabolite ratios.

### 2.4. Statistics

All statistical analyses were performed using the statistical software R (3.0.2) [37]. In a first step, we graphically screened for outliers and normality. An absolute metabolite concentration that lay greater than 1 standard deviation (SD) away from its nearest neighbor was considered to be an outlier and this measurement was excluded from the analysis. Principal component analysis score plots were used as a complementary tool to ensure that no outliers remained undetected.

Differences in clinical parameters between baseline and follow-up were calculated using the paired Wilcoxon rank test. Associations between markers for insulin and glucohomeostasis were quantified using Spearman rank correlation coefficients.

The changes in the clinical markers, metabolite concentrations, and metabolite ratios over the one-year intervention are expressed as the relative difference of baseline and follow-up measurements (with the baseline values being the reference). For each time point (baseline and follow-up) as well as for the relative change, we calculated the following model to assess the association between the metabolites and the clinical parameters: (1) firstly, in order to account for the effect of obesity status on the metabolite level, we fitted age and sex adjusted robust regression models of the BMI on the metabolite using the M-estimator with Huber bisquare weighting (R package MASS); (2) subsequently, we regressed the obtained metabolite residuals on markers for IR with robust regression models using the M-estimator with Huber bisquare weighting (R package MASS). *p* values and estimates are taken as proxies for the strengths and directions of the associations. Results of selected clinical outcomes are represented graphically in Manhattan plots, where the log_10_⁡(*p*) values are plotted and the sign is used to indicate the direction of the relationship, as assessed by the robust regression model. Due to the small sample size and in order not to veil differences in *p* values, we will report the raw (unadjusted) *p* values. The significance level was thus set at *p* < 0.05. Bonferroni corrected *p* values can be obtained by multiplying the reported *p* values with the factor 42 (number of analytes tested). The Bonferroni corrected significance level is 0.0012.

## 3. Results

### 3.1. Population Characteristics

Characteristics of participating children are presented in Table 1. In all obese children, waist circumference and 2-hour oGTT glucose decreased significantly during the intervention period. Additionally, insulin levels and HOMA decreased in the group of 40 obese children with substantial weight loss. In contrast, insulin levels and HOMA increased in the group of obese children without substantial weight loss. Since HOMA and insulin were strongly correlated (Table 2) and HbA1C and fasting glucose showed no changes between the two time points in any of the groups, in contrast to HOMA, waist circumference, and 2 h glucose in oGTT (Table 1), we focused our data analysis on HOMA, 2-hour oGTT glucose, and waist circumference. Waist circumference showed no significant association with the metabolites. No difference between puberty and HOMA status was found at baseline (*p* = 0.44), but after the intervention period (*p* = 0.036) with pubertal children having higher HOMA values.

Associations of all clinical parameters and metabolites are reported in the Supplementary Material (available online at http://dx.doi.org/10.1155/2016/2108909).

### 3.2. HOMA

**Figure 1:**
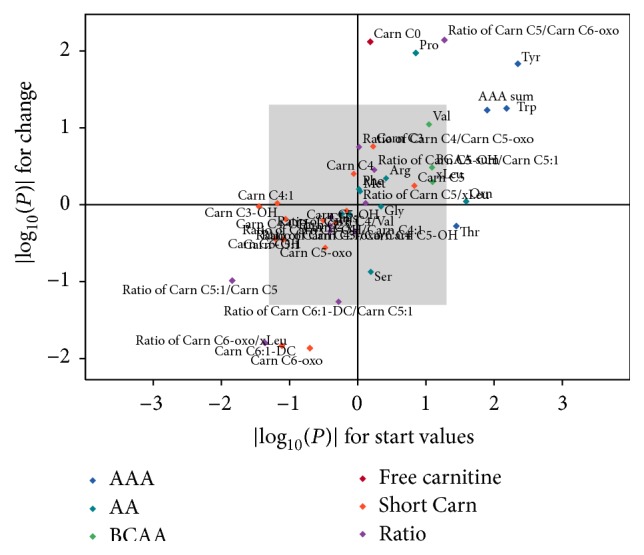
Associations of amino acids (AA) and acylcarnitines (Carn) with HOMA. Associations were calculated at baseline (*x*-axis) and for changes of AA and Carn to changes of HOMA during the intervention (*y*-axis) period in all children (*n* = 80). Displayed are the absolute log⁡(*p*) values of the applied obesity-independent robust regression models for both associations. AAA, aromatic amino acids; BCAA, branched-chain amino acids.

**Figure 2:**
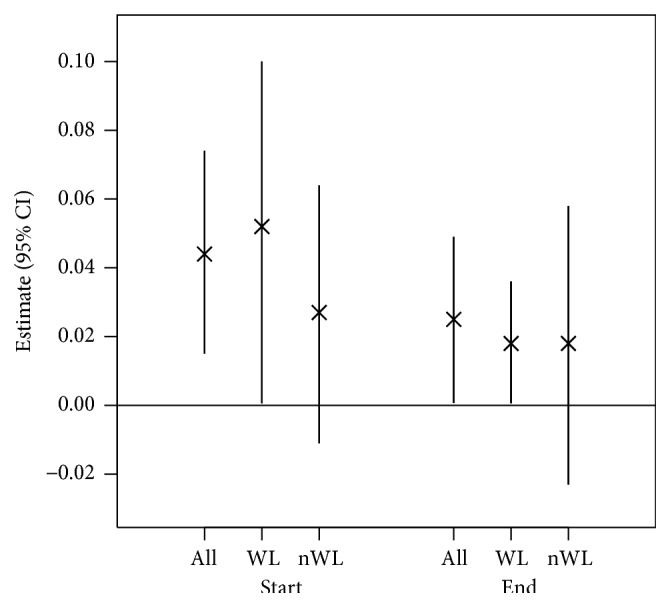
Estimates (95% CI) for associations of tyrosine with HOMA. Associations were calculated at baseline (Start) and after the intervention (End). Associations were calculated for all obese children (*n* = 80), children with substantial weight loss (WL, *n* = 40), and children without substantial weight loss (nWL, *n* = 40). 95% CI: 95% confidence interval.

**Figure 3:**
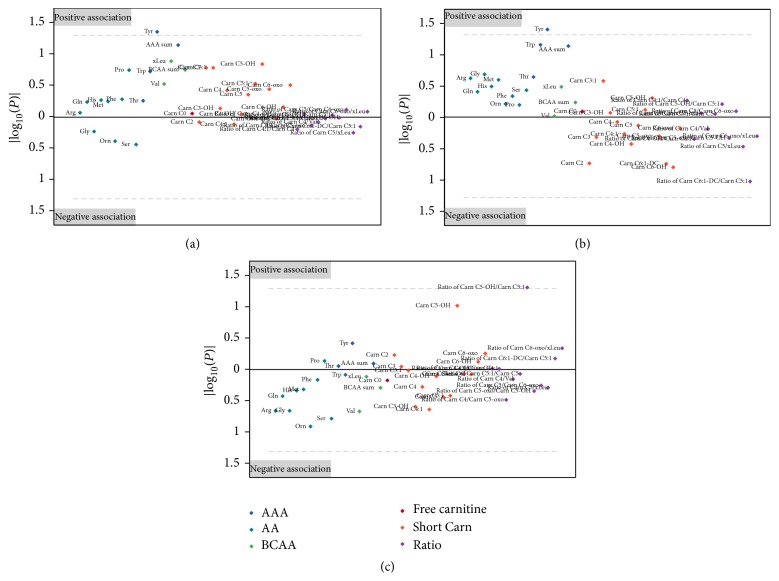
Manhattan plot for associations of amino acids (AA) and acylcarnitines (Carn) with HOMA after the one-year lifestyle intervention for all obese children (a), children with substantial weight loss (b), and children without substantial weight loss (c). Plotted are the log_10_⁡(*p*) values and the sign is used to indicate the direction of the relationship, as assessed by the robust regression model. The area below or above the dashed lines contains metabolites that are significantly related to HOMA (*p* values < 0.05). AAA, aromatic amino acids; BCAA, branched-chain amino acids.

At baseline, HOMA was positively associated with Tyr (*p* = 0.004, Figure 2), Trp (*p* = 0.007), sum of AAA (*p* = 0.013), ornithine (Orn, *p* = 0.026), and threonine (Thr, *p* = 0.036) and negatively associated with Carn C3-OH (*p* = 0.036) and the ratios of Carn C5:1/Carn C5 (*p* = 0.014) and Carn C6-oxo/xLeu (*p* = 0.044) in all obese children (Figure 1). After the end of the intervention, only Tyr was associated with HOMA in all obese children (*p* = 0.044, Figures 2 and 3). In a stratified analysis including the 40 children with substantial weight loss, HOMA was negatively associated with the ratio of Carn C6-oxo/xLeu (*p* = 0.011), Carn C6-oxo (*p* = 0.023), and Carn C4 (*p* = 0.031) and positively associated with Carn C4/Carn C5-oxo (*p* = 0.041) and Tyr (*p* = 0.047, Figure 2) at baseline. After the intervention, only Tyr was associated with HOMA (*p* = 0.041) in the children with substantial weight loss (Figures 2 and 3). Children without substantial weight loss showed different associations for HOMA. Thr (*p* < 0.001) and proline (Pro, *p* = 0.0322) were positively associated with HOMA, while the ratios of Carn C5:1/Carn C5 (*p* = 0.030) and Carn C4/Val (*p* = 0.033) were negatively associated at baseline. After the intervention, only the ratio of Carn C5-OH/Carn C5:1 was associated with HOMA (*p* = 0.048) in children without substantial weight loss (Figure 3). The significant associations between the relative change of HOMA during the intervention period and the relative change of AA and Carn are shown in Table 3 for all obese children (Figure 1), children with substantial weight loss, and children without substantial weight loss. The change of ratio of Carn C5/Carn C6-oxo was positively associated with change of HOMA in all three groups, while this was true for the ratio of Carn C4/Carn C5-oxo only in children with substantial weight loss. Changes of Tyr were again positively correlated with changes in HOMA in all children and children with substantial weight loss.

### 3.3. Two-Hour oGTT Glucose

Two-hour oGTT glucose showed different associations compared to HOMA, particularly in children with substantial weight loss. At baseline, the ratios of Carn C4:1/Carn C4 (*p* = 0.011, negative), Carn C4/Carn C5-oxo (*p* = 0.023, positive), and Carn C4/Val (*p* = 0.05, positive) as well as histidine (His, *p* = 0.040, negative), serine (Ser, *p* = 0.043, negative), and Carn C4 (*p* = 0.049, positive) were associated with 2-hour oGTT glucose in children with substantial weight loss, while children without substantial weight loss showed only positive associations between arginine (Arg) and 2-hour oGTT glucose at baseline. After the intervention, 2-hour oGTT glucose was associated negatively with Ser (*p* = 0.048) and Orn (*p* = 0.039) in children with substantial weight loss and with glutamine (Gln, *p* = 0.011) and Carn C3-OH (*p* = 0.012) in children without substantial weight loss. Interestingly, changes of 2-hour oGTT glucose during the one-year intervention period were not significantly associated with changes of any of the measured metabolites, ratios, or sums in any of the groups.

## 4. Discussion

To our knowledge, this is the first longitudinal study analysing the relationships between metabolites and markers of glucose metabolism in obese children. Tyr is the only metabolite which was significantly associated with HOMA at baseline and after intervention. Changes of Tyr over time were also positively associated with changes of HOMA in our obesity-independent model. Thus, Tyr, rather than BCAA, seems to be associated with IR. This is in accordance with a recent study where Tyr was identified as the most important metabolite in a random forest analysis in obese children [26]. In the same study, a combination of BCAA and AAA was most strongly related to HOMA. Furthermore, Tyr was found to be a strong predictor for diabetes in South Asian men [21]. Tyr is biosynthesised endogenously by hydroxylation of Phe by phenylalanine hydroxylase in mammals [38]. Since Phe was not associated with HOMA in any of the groups at any time point, we assume that there is no confounding dietary effect on Tyr levels. Tyr stimulates insulin secretion, but other AAs are more effective in stimulating insulin release [39]. In contrast, Michaliszyn et al. showed a positive association of *β*-cell function and all AAs except for Tyr and citrulline in adolescents [40]. The same group reported lower AA plasma levels, except for Tyr, in diabetic adolescents [41], which is in contrast to the role of elevated AA in IR [42]. However, the effect on insulin secretion should not be driving the relation between Tyr and HOMA. An influence on Tyr metabolism appears more likely. Insulin is known to increase tyrosine aminotransferase (TAT) activity in rat liver [43], probably by selectively slowing down the rate of degradation of TAT [44]. TAT catalyses the transamination of Tyr to p-hydroxyphenylpyruvate. This should result in lower Tyr levels. A possible inhibition of TAT may occur due to higher Cys levels. Cys is an inhibitor of TAT [19, 45]. In a recent study, only Cys and Tyr were found to be increased in nonobese adults who had one symptom of the metabolic syndrome [14]. With further progression of the metabolic syndrome, BCAA and Phe were enhanced in subjects with two or more symptoms of the metabolic syndrome. It seems that alterations in Cys and Tyr metabolism precede changes in BCAA metabolism. Thus, Tyr is a potential early marker for the onset of IR. Higher insulin levels in the IR state may still cover demands to ensure adequate glucose metabolism, but Tyr may be affected and Tyr may present an early biomarker for the onset of IR in obese children. This predictive value of Tyr was shown in previous studies in adults, along with BCAA in young adults [16, 18]. In the presented study, we could not investigate Tyr as predictor for later IR, since the “Obeldicks” study has an interventional design not a prospective one. Contrarily, Lee et al. found BCAA, and not AAA, as predictive marker for IR in Korean children [27]. Thus, further prospective, longitudinal studies are required to unravel associations between AA and IR with respect to sex and ethnicity.

Furthermore, Tyr can affect BCAA levels, since BCAA and AA compete for the same neutral AA transporter for cellular uptake [46]. Thus, prolonged elevated Tyr levels may also result in elevated BCAA level. Many studies found altered levels of BCAA when studying obesity in adults [9], but also when looking at IR and T2DM in adults [13, 15–18, 42]. Similar results were found in a few cross-sectional studies in children [12, 25]. We found no association between HOMA and BCAA, neither at baseline nor after the intervention. A previous cross-sectional metabolomic analysis of the presented population did not find different BCAA levels between obese and normal weight children [47]. Levels of BCAA, being essential AAs, are mainly defined by the diet. Thus, higher BCAA levels later in life may result from competition for the neutral AAs transport [46], higher protein intake, and/or disturbed BCAA clearance [48]. During BCAA degradation, Val, Leu, and Ile are first degraded to the *α*-keto acids C5-oxo and C6-oxo by the branched-chain amino transferase (BCAT). These keto acids are subsequently reduced by branched-chain *α*-keto acid dehydrogenase (BCKDH) in the rate limiting step [49]. To identify alterations of BCAA metabolism in obesity and IR, we calculated the ratios of the different steps in the BCAA degradation pathway. We found that the ratio of C6-oxo to C5 was positively associated with HOMA at baseline and in the change during the intervention period in all three groups. The ratio of C5-oxo to C4 was positively associated with HOMA only in children with substantial weight loss only. Both ratios are markers for the second, rate limiting degradation step of BCAA which is regulated by BCAA itself to keep BCAA concentrations at a constant level. Thus, this pathway may have been upregulated in our study resulting in BCAA levels not associated with HOMA. In contrast, all other ratios showed no significant association with HOMA or were negatively associated with HOMA, particularly the first step of xLeu degradation to methyl-ketopentanoate (C6-oxo) by BCAT. It was shown recently that IR subjects have a significant reduction in BCAT expression and other enzymes involved in BCAA metabolism in the adipose tissue compared to none-IR subjects [50]. Additionally, BCAT2 (mitochondrial) was significantly downregulated, whereas BCAT1 (cytosolic) was significantly upregulated in the adipose tissue of obese subjects [51]. Other mitochondrial genes of BCAA metabolism were also downregulated in adipose tissue, but not in liver or muscle tissue. Our study showed that the reduction of BCAA degradation seems to precede elevated BCAA levels in IR state or obesity in children, since no elevated BCAA were found but alterations in BCAA metabolite ratios. Thus, insulin negatively alters BCAA metabolism resulting in higher plasma BCAA in later life, when BCAA concentrations overcome their own degradation and contribute to the vicious circle of IR [52]. The analysis of metabolite profiles in children allowed us to study this early development effect of IR, but far more studies in children and early adulthood are needed to investigate the molecular changes in the early state of obesity and IR. However, BCAA and BCAA metabolism are more affected by protein-rich diet than other AAs [53], and thus diet is a known confounder, especially in obese patients with higher protein intake [15, 22]. After the intervention, in a state of homogenous lifestyle and diet, the BCAA ratios were no longer significantly associated with HOMA, except for the ratio of Carn C5-OH to Carn C5:1, which was positively associated with HOMA in children without substantial weight loss. Thus, lifestyle may have strong effect on BCAA metabolism, and the results found at baseline were hidden by the homogenous lifestyle of the studied cohort. This possible confounding effect of diet and lifestyle on BCAA metabolism has to be investigated in further studies. The nonsignificant association of HOMA and BCAA could also be result of less power of our study. Tai et al. depicted the same challenge when they found an association of IR with BCAA in a large group of Chinese men, but not in a smaller group of Indian Asian men [15]. Two-hour oGTT glucose showed different associations with AA and AA derivatives compared to HOMA. Particularly, Ser was negatively associated with 2-hour oGTT glucose. Since fewer studies exist on relations of 2-hour oGTT glucose to metabolites, we can only speculate about the underlying mechanisms. Ser and glycine (Gly) were found to be decreased in obese Hispanic children [26] and in obese Korean children [27]. The concentrations of Gly and Ser were found to be lower in diabetic than in fasted normal rats [54]. The authors concluded that the contribution of Ser to gluconeogenesis becomes proportionally higher in diabetes. Thus, an increased gluconeogenesis rate in diabetic or prediabetic patients most likely leads to decreased Ser levels. In this case, Ser seems to be the AA which is first affected by higher gluconeogenesis rate. But to unravel this relation, further studies are needed. Another explanation is the use of consumed Ser for SM synthesis, since SM are elevated in IR state [55]. Furthermore, we have to recall the relatively low reliability of 2 h glucose in oGTT [56]. Additionally, elevated 2 h glucose levels in obese children tend to normalize in follow-up even without weight loss as also demonstrated in our study [57, 58].

All the described relations between metabolites and HOMA or 2-hour oGTT glucose were driven by children with substantial weight loss. Children without substantial weigh loss showed fewer and different associations. Thus, it is plausible that different metabolomic changes are associated with different types of IR. However, HOMA and 2-hour oGTT glucose did not change significantly in children without substantial weight loss during the intervention period, and thus the information in these parameters may be too little to depict associations between metabolites and clinical parameters in this group. Nevertheless, estimates for the associations between metabolites and HOMA were different at baseline, in change, and at follow-up, assuming different metabolic pattern which could be related to IR, the intervention, or weight loss. Thus, further prospective studies should focus on the relation of IR to different metabolomic patterns.

However, we were not able to differentiate the effect of diet, increased physical exercise, and weight loss on metabolites and their ratio concentrations due to our study protocol. Unfortunately, we could not perform stratified analyses to the influence of pubertal status on the associations of metabolites with IR. An explorative statistical approach showed no influence on the association of IR with Tyr, but on Carn. Further studies with larger sample number are required to determine differences in IR development with respect to puberty status. A limitation of our study is that BMI percentiles were used to classify overweight. Although BMI is a good measure for overweight, it is not a precise measure of body fat mass. Furthermore, the degree of obesity was relatively homogeneous in our obese children. Additionally, the HOMA model is only an assessment of IR [59]. Clamp studies are actually the gold standard for analysing IR. In addition to the small sample number, these facts reduced the odds to detect associations between metabolites and clinical outcomes. The relatively low sample number also reduced the statistical power of the presented data analysis, which kept us from correction for multiple testing. Among the strengths of our study are the longitudinal design and the analysed children that were naïve to drugs and other diseases and had similar lifestyle during the one-year intervention. The additional focus on ratios allowed for a closer insight into degradation pathways associated with obesity-related IR. Thus, the influence of diet and physical activity on changes of metabolite levels should be limited.

## 5. Conclusions

This study provides novel insights into the longitudinal interrelations of IR and obesity markers to metabolites and generates possible questions for further mechanistic studies of IR in obese children. Our cross-sectional and longitudinal analyses confirm a relationship between the Tyr and HOMA in obese children. So, Tyr and the Tyr metabolism should be focused more on in studies searching for early biomarkers and predictors in the switch from obesity to IR. In contrast, BCAA levels were negatively related to IR in cross-sectional analyses, while there was no significant association in the longitudinal analysis, which does not support a causal role of BCAA in inducing IR. Furthermore, responders to the intervention showed different associations between HOMA and AA compared to nonresponders, which appears to reflect different mechanisms for the development of obesity-induced IR. Further studies should also explore other analytes which were not determined in our study, such as p-hydroxyphenylpyruvate, fumarate, or acetoacetate that are involved in Tyr metabolism and sulfur containing AA.

## Supplementary Material

The supplementary material contains the estimates, 95% confidence intervals (95%CI), and *p*-values (*p*) of the estimate for the associations of all metabolites to all clinical parameters. The first table depicts the associations at baseline, the second one the associations in the changes from baseline to the end of the intervention, and the third table contains the associations at the end of the intervention. Each association is calculated for the whole cohort (_all) and stratified for children with (_WL) and without (_nWL) substantial weight loss during the intervention. Estimates, CIs, and *p*-values were calculated with robust regression models.

## Figures and Tables

**Table 1:** Characteristics of participating children at start and end point of the 1-year intervention period. Characteristics are shown for all obese children (*n* = 80), children with substantial weight loss (WL, *n* = 40), and children without substantial weight loss (nWL, *n* = 40) as mean ± SD unless stated otherwise.

Parameter	All start (%)	All end (%)	WL start (%)	WL end (%)	nWL start (%)	nWL end (%)
Sex, male	36 (45%)		18 (45%)		18 (45%)	
Age (years)	11.5 ± 2.42	12.5 ± 2.42	10.6 ± 2.54	11.6 ± 2.54	12.4 ± 1.9	13.4 ± 1.9
Prepubertal	34 (42%)	26 (32%)	23 (58%)	18 (45%)	11 (28%)	8 (20%)
Early pubertal	42 (52%)	44 (55%)	17 (42%)	22 (55%)	25 (62%)	22 (55%)
Postpubertal	4 (5%)	10 (12%)	—	—	4 (10%)	10 (25%)
BMI-SDS	2.4 ± 0.45	2.1 ± 0.63^*∗*^	2.4 ± 0.44	1.7 ± 0.58^*∗*^	2.4 ± 0.46	2.4 ± 0.47^*∗*^
Waist circumference (cm)	91.7 ± 14	89.3 ± 13.89^*∗*^	87 ± 13.59	81 ± 10.99^*∗*^	96.5 ± 12.88	97.3 ± 11.55
Insulin (mU/L)	19.9 ± 15.01	17 ± 12.39	18 ± 12.01	9.3 ± 3.87^*∗*^	21.9 ± 17.44	25.1 ± 13.14^*∗*^
Fasting glucose (mg/dL)	86.3 ± 7.38	87.1 ± 6.39	84.8 ± 7.05	85.5 ± 5.76	87.8 ± 7.47	88.8 ± 6.65
2-hour oGTT glucose (mg/dL)	132.7 ± 25.02	113.9 ± 23.94^*∗*^	133.9 ± 24.26	98.9 ± 10.42^*∗*^	131.4 ± 25.99	128.9 ± 24.28
HbA1C (mmol/mol Hb)	373.43 ± 32.92	372.81 ± 37.98	364.16 ± 30.12	363.77 ± 30.81	382.95 ± 33.32	382.61 ± 42.78
HOMA	4.29 ± 3.1	3.79 ± 3.2	4.01 ± 3	1.96 ± 0.81^*∗*^	4.58 ± 3.21	5.67 ± 3.64^*∗*^

^*∗*^Significant different means between start and end point (*p* < 0.05, paired Wilcoxon rank sum test).

**Table 2:** Spearman correlation coefficients of markers of insulin homeostasis in all obese children (*n* = 80) at baseline.

	Fasting glucose (mg/dL)	2-hour oGTT glucose (mg/dL)	HbA1C (mmol/mol Hb)	Insulin (mU/L)	HOMA	BMI-SDS	Waist circumference (cm)
Fasting glucose (mg/dL)	1	0.246	0.231	0.091	0.154	−0.001	0.241
2-hour oGTT glucose (mg/dL)		1	0.188	0.113	0.135	0.153	0.164
HbA1C (mmol/mol Hb)			1	0.120	0.176	0.116	0.234
Insulin (mU/L)				1	0.984	0.343	0.586
HOMA					1	0.402	0.669
BMI-SDS						1	0.445
Waist circumference (cm)							1

**Table 3:** Estimates and *p* values (*p*) of changes in metabolite concentrations which are significantly associated with changes in HOMA in at least one (sub)group (All, WL, and nWL). Change is defined as the relative change over the one-year intervention. Estimates are given with 95% confidence interval (CI). Estimates, confidence intervals, and *p* values were calculated with robust regression models. WL, children with substantial weight loss; nWL, children without substantial weight loss; AAA, aromatic amino acids; Carn, acylcarnitine; Pro, proline; Trp, tryptophan; Tyr, tyrosine; Val, valine, xLeu, sum of leucine and isoleucine.

Analyte	All (*n* = 80)		WL (*n* = 40)		nWL (*n* = 40)	
	Estimate [95% CI]	*p*	Estimate [95% CI]	*p*	Estimate [95% CI]	*p*
AAA sum	0.79 [−0.04; 1.60]	0.059	1.04 [0.29; 1.80]	0.009	0.27 [−1.40; 1.90]	0.742
Carn C0	1.10 [0.29; 1.90]	0.008	1.71 [0.88; 2.50]	<0.001	0.24 [−1.30; 1.70]	0.751
Carn C3	0.34 [−0.16; 0.83]	0.174	0.49 [0.03; 0.96]	0.036	0.24 [−0.72; 1.20]	0.615
Carn C6:1-DC	−0.33 [−0.59; −0.06]	0.015	−0.29 [−0.47; −0.10]	0.003	−0.37 [−1.10; 0.38]	0.342
Carn C6-oxo	−0.24 [−0.43; −0.05]	0.014	−0.21 [−0.35; −0.08]	0.003	−0.21 [−0.73; 0.31]	0.438
Pro	0.81 [0.19; 1.40]	0.011	0.72 [0.11; 1.30]	0.023	0.88 [−0.08; 1.80]	0.067
Ratio of Carn C4/Carn C5-oxo	0.23 [−0.09; 0.55]	0.177	0.48 [0.18; 0.77]	0.003	−0.21 [−0.86; 0.45]	0.530
Ratio of Carn C5/Carn C6-oxo	0.24 [0.07; 0.41]	0.007	0.22 [0.08; 0.35]	0.002	0.29 [0.02; 0.57]	0.041
Ratio of Carn C6:1-DC/Carn C5:1	−0.27 [−0.54; 0.01]	0.055	−0.22 [−0.41; −0.03]	0.024	−0.39 [−1.10; 0.37]	0.321
Ratio of Carn C6-oxo/xLeu	−0.19 [−0.34; −0.03]	0.016	−0.15 [−0.25; −0.04]	0.007	−0.24 [−0.84; 0.37]	0.442
Trp	0.93 [−0.02; 1.90]	0.056	1.13 [0.14; 2.10]	0.027	0.44 [−1.30; 2.20]	0.613
Tyr	0.79 [0.17; 1.40]	0.015	1.09 [0.51; 1.70]	0.001	0.28 [−0.99; 1.50]	0.651
Val	0.65 [−0.11; 1.40]	0.090	0.73 [0.07; 1.40]	0.033	0.48 [−1.10; 2.10]	0.544

## References

[1] Pulgarón E. R. (2013). Childhood obesity: a review of increased risk for physical and psychological comorbidities. *Clinical Therapeutics*.

[2] Csábi G., Török K., Molnár D., Jeges S. (2000). Presence of metabolic cardiovascular syndrome in obese children. *European Journal of Pediatrics*.

[3] Freedman D. S., Dietz W. H., Srinivasan S. R., Berenson G. S. (1999). The relation of overweight to cardiovascular risk factors among children and adolescents: the Bogalusa Heart study. *Pediatrics*.

[4] Reinehr T., Andler W., Denzer C., Siegried W., Mayer H., Wabitsch M. (2005). Cardiovascular risk factors in overweight German children and adolescents: relation to gender, age and degree of overweight. *Nutrition, Metabolism and Cardiovascular Diseases*.

[5] Biro F. M., Wien M. (2010). Childhood obesity and adult morbidities. *The American Journal of Clinical Nutrition*.

[6] Reinehr T., de Sousa G., Andler W. (2005). Longitudinal analyses among overweight, insulin resistance, and cardiovascular risk factors in children. *Obesity Research*.

[7] Coughlin S. S. (2014). Toward a road map for global -omics: a primer on -omic technologies. *American Journal of Epidemiology*.

[8] Sales N. M. R., Pelegrini P. B., Goersch M. C. (2014). Nutrigenomics: definitions and advances of this new science. *Journal of Nutrition and Metabolism*.

[9] Rauschert S., Uhl O., Koletzko B., Hellmuth C. (2014). Metabolomic biomarkers for obesity in humans: a short review. *Annals of Nutrition and Metabolism*.

[10] Demine S., Reddy N., Renard P., Raes M., Arnould T. (2014). Unraveling biochemical pathways affected by mitochondrial dysfunctions using metabolomic approaches. *Metabolites*.

[11] Lustgarten M. S., Lyn Price L., Phillips E. M., Fielding R. A. (2013). Serum glycine is associated with regional body fat and insulin resistance in functionally-limited older adults. *PLoS ONE*.

[12] Perng W., Gillman M. W., Fleisch A. F. (2014). Metabolomic profiles and childhood obesity. *Obesity*.

[13] Newgard C. B., An J., Bain J. R. (2009). A branched-chain amino acid-related metabolic signature that differentiates obese and lean humans and contributes to insulin resistance. *Cell Metabolism*.

[14] Mohorko N., Petelin A., Jurdana M., Biolo G., Jenko-Pražnikar Z. (2015). Elevated serum levels of cysteine and tyrosine: early biomarkers in asymptomatic adults at increased risk of developing metabolic syndrome. *BioMed Research International*.

[15] Tai E. S., Tan M. L. S., Stevens R. D. (2010). Insulin resistance is associated with a metabolic profile of altered protein metabolism in Chinese and Asian-Indian men. *Diabetologia*.

[16] Wurtz P., Soininen P., Kangas A. J. (2013). Branched-chain and aromatic amino acidsare predictors of insulinresistance in young adults. *Diabetes Care*.

[17] Fiehn O., Garvey W. T., Newman J. W., Lok K. H., Hoppel C. L., Adams S. H. (2010). Plasma metabolomic profiles reflective of glucose homeostasis in non-diabetic and type 2 diabetic obese African-American women. *PLoS ONE*.

[18] Shah S. H., Crosslin D. R., Haynes C. S. (2012). Branched-chain amino acid levels are associated with improvement in insulin resistance with weight loss. *Diabetologia*.

[19] Adams S. H. (2011). Emerging perspectives on essential amino acid metabolism in obesity and the insulin-resistant state. *Advances in Nutrition*.

[20] Chen H.-H., Tseng Y. J., Wang S.-Y. (2015). The metabolome profiling and pathway analysis in metabolic healthy and abnormal obesity. *International Journal of Obesity*.

[21] Tillin T., Hughes A. D., Wang Q. (2015). Diabetes risk and amino acid profiles: cross-sectional and prospective analyses of ethnicity, amino acids and diabetes in a South Asian and European cohort from the SABRE (Southall And Brent REvisited) Study. *Diabetologia*.

[22] Würtz P., Mäkinen V.-P., Soininen P. (2012). Metabolic signatures of insulin resistance in 7,098 young adults. *Diabetes*.

[23] Villarreal-Pérez J., Villarreal-Martínez J., Lavalle-González F. (2014). Plasma and urine metabolic profiles are reflective of altered beta-oxidation in non-diabetic obese subjects and patients with type 2 diabetes mellitus. *Diabetology & Metabolic Syndrome*.

[24] Wang T. J., Larson M. G., Vasan R. S. (2011). Metabolite profiles and the risk of developing diabetes. *Nature Medicine*.

[25] Newbern D., Balikcioglu P. G., Balikcioglu M. (2014). Sex differences in biomarkers associated with insulin resistance in obese adolescents: metabolomic profiling and principal components analysis. *Journal of Clinical Endocrinology and Metabolism*.

[26] Butte N. F., Liu Y., Zakeri I. F. (2015). Global metabolomic profiling targeting childhood obesity in the Hispanic population. *The American Journal of Clinical Nutrition*.

[27] Lee A., Jang H. B., Ra M. (2014). Prediction of future risk of insulin resistance and metabolic syndrome based on Korean boy's metabolite profiling. *Obesity Research & Clinical Practice*.

[28] Mccormack S. E., Shaham O., Mccarthy M. A. (2013). Circulating branched-chain amino acid concentrations are associated with obesity and future insulin resistance in children and adolescents. *Pediatric Obesity*.

[29] Reinehr T., de Sousa G., Toschke A. M., Andler W. (2006). Long-term follow-up of cardiovascular disease risk factors in children after an obesity intervention. *American Journal of Clinical Nutrition*.

[30] Reinehr T., Hinney A., de Sousa G., Austrup F., Hebebrand J., Andler W. (2007). Definable somatic disorders in overweight children and adolescents. *The Journal of Pediatrics*.

[31] Reinehr T., Kiess W., Kapellen T., Andler W. (2004). Insulin sensitivity among obese children and adolescents, according to degree of weight loss. *Pediatrics*.

[32] Reinehr T., Wolters B., Knop C. (2015). Changes in the serum metabolite profile in obese children with weight loss. *European Journal of Nutrition*.

[33] Cole T. J. (1990). The LMS method for constructing normalized growth standards. *European Journal of Clinical Nutrition*.

[34] Kromeyer-Hauschild K., Wabitsch M., Kunze D. (2001). Perzentile für den Body-mass-Index für das Kindes- und Jugendalter unter Heranziehung verschiedener deutscher Stichproben. *Monatsschrift Kinderheilkunde*.

[35] American Diabetes Association (2000). Type 2 diabetes in children and adolescents. *Diabetes Care*.

[36] Matthews D. R., Hosker J. P., Rudenski A. S., Naylor B. A., Treacher D. F., Turner R. C. (1985). Homeostasis model assessment: insulin resistance and *β*-cell function from fasting plasma glucose and insulin concentrations in man. *Diabetologia*.

[37] http://www.r-project.org/.

[38] Woo S. L. C., Lidsky A. S., Guttler F., Chandra T., Robson K. J. (1983). Cloned human phenylalanine hydroxylase gene allows prenatal diagnosis and carrier detection of classical phenylketonuria. *Nature*.

[39] Kuhara T., Ikeda S., Ohneda A., Sasaki Y. (1991). Effects of intravenous infusion of 17 amino acids on the secretion of GH, glucagon, and insulin in sheep. *American Journal of Physiology—Endocrinology and Metabolism*.

[40] Michaliszyn S. F., Sjaarda L. A., Mihalik S. J. (2012). Metabolomic profiling of amino acids and *β*-cell function relative to insulin sensitivity in youth. *The Journal of Clinical Endocrinology & Metabolism*.

[41] Mihalik S. J., Michaliszyn S. F., de las Heras J. (2012). Metabolomic profiling of fatty acid and amino acid metabolism in youth with obesity and type 2 diabetes: evidence for enhanced mitochondrial oxidation. *Diabetes Care*.

[42] Huffman K. M., Shah S. H., Stevens R. D. (2009). Relationships between circulating metabolic intermediates and insulin action in overweight to obese, inactive men and women. *Diabetes Care*.

[43] Labrie F., Korner A. (1969). Effect of glucagon, insulin, and thyroxine on tyrosine transaminase and tryptophan pyrrolase of rat liver. *Archives of Biochemistry and Biophysics*.

[44] Spencer C. J., Heaton J. H., Gelehrter T. D., Richardson K. I., Garwin J. L. (1978). Insulin selectively slows the degradation rate of tyrosine aminotransferase. *Journal of Biological Chemistry*.

[45] Hargrove J. L., Trotter J. F., Ashline H. C., Krishnamurti P. V. (1989). Experimental diabetes increases the formation of sulfane by transsulfuration and inactivation of tyrosine aminotransferase in cytosols from rat liver. *Metabolism*.

[46] Fernstrom J. D. (2005). Branched-chain amino acids and brain function. *Journal of Nutrition*.

[47] Wahl S., Yu Z., Kleber M. (2012). Childhood obesity is associated with changes in the serum metabolite profile. *Obesity Facts*.

[48] Marchesini G., Bianchi G. P., Vilstrup H., Capelli M., Zoli M., Pisi E. (1991). Elimination of infused branched-chain amino-acids from plasma of patients with non-obese type 2 diabetes mellitus. *Clinical Nutrition*.

[49] Brosnan J. T., Brosnan M. E. (2006). Branched-chain amino acids: enzyme and substrate regulation. *Journal of Nutrition*.

[50] Serralde-Zúñiga A. E., Guevara-Cruz M., Tovar A. R. (2014). Omental adipose tissue gene expression, gene variants, branched-chain amino acids, and their relationship with metabolic syndrome and insulin resistance in humans. *Genes and Nutrition*.

[51] Mardinoglu A., Kampf C., Asplund A. (2014). Defining the human adipose tissue proteome to reveal metabolic alterations in obesity. *Journal of Proteome Research*.

[52] O'Connell T. M. (2013). The complex role of branched chain amino acids in diabetes and cancer. *Metabolites*.

[53] Kirchberg F. F., Harder U., Weber M. (2015). Dietary protein intake affects amino acid and acylcarnitine metabolism in infants aged 6 months. *The Journal of Clinical Endocrinology & Metabolism*.

[54] Hetenyi G., Anderson P. J., Raman M., Ferrarotto C. (1988). Gluconeogenesis from glycine and serine in fasted normal and diabetic rats. *Biochemical Journal*.

[55] Gill J. M. R., Sattar N. (2009). Ceramides: a new player in the inflammation-insulin resistance paradigm?. *Diabetologia*.

[56] Libman I. M., Barinas-Mitchell E., Bartucci A., Robertson R., Arslanian S. (2008). Reproducibility of the oral glucose tolerance test in overweight children. *Journal of Clinical Endocrinology and Metabolism*.

[57] Kleber M., deSousa G., Papcke S., Wabitsch M., Reinehr T. (2011). Impaired glucose tolerance in obese white children and adolescents: three to five year follow-up in untreated patients. *Experimental and Clinical Endocrinology and Diabetes*.

[58] Kleber M., Lass N., Papcke S., Wabitsch M., Reinehr T. (2010). One-year follow-up of untreated obese white children and adolescents with impaired glucose tolerance: high conversion rate to normal glucose tolerance. *Diabetic Medicine*.

[59] Uwaifo G. I., Fallon E. M., Chin J., Elberg J., Parikh S. J., Yanovski J. A. (2002). Indices of insulin action, disposal, and secretion derived from fasting samples and clamps in normal glucose-tolerant black and white children. *Diabetes Care*.

